# Aggregation-Induced Emission (AIE)-Labeled Cellulose Nanocrystals for the Detection of Nitrophenolic Explosives in Aqueous Solutions

**DOI:** 10.3390/nano9050707

**Published:** 2019-05-07

**Authors:** Xiu Ye, Haoying Wang, Lisha Yu, Jinping Zhou

**Affiliations:** Department of Chemistry, Engineering Research Center of Natural Polymer-based Medical Materials in Hubei Province, and Key Laboratory of Biomedical Polymers of Ministry of Education, Wuhan University, Wuhan 430072, China; yx444131997@gmail.com (X.Y.); wang159hy@gmail.com (H.W.); LisaYu12@whu.edu.cn (L.Y.)

**Keywords:** cellulose nanocrystals, aggregation-induced emission (AIE), detection, nitrophenolic explosives, fluorescence

## Abstract

Aggregation-induced emission (AIE) active cellulose nanocrystals (TPE-CNCs) were synthesized by attaching tetraphenylethylene (TPE) to cellulose nanocrystals (CNCs). The structure and morphology of TPE-CNCs were characterized by FT-IR, XRD, ζ-potential measurements, elemental analysis, TEM, atomic force microscopy (AFM), and dynamic laser light scattering (DLS). Fluorescent properties of TPE-CNCs were also further studied. Unlike aggregation-caused quenching (ACQ), TPE-CNCs emitted weak fluorescence in the dilute suspensions, while emitting efficiently in the aggregated states. The AIE mechanism of TPE-CNCs was attributed to the restriction of an intramolecular rotation (RIR) process in the aggregated states. TPE-CNCs displayed good dispersity in water and stable fluorescence, which was reported through the specific detection of nitrophenolic explosives in aqueous solutions by a fluorescence quenching assay. The fluorescence emissions of TPE-CNCs showed quantitative and sensitive responses to picric acid (PA), 2,4-dinitro-phenol (DNP), and 4-nitrophenol (NP), and the detection limits were 220, 250, and 520 nM, respectively. Fluorescence quenching occurred through a static mechanism via the formation of a nonfluorescent complex between TPE-CNCs and nitrophenolic analytes. A fluorescence lifetime measurement revealed that the quenching was a static process. The results demonstrated that TPE-CNCs were excellent sensors for the detection of nitrophenolic explosives in aqueous systems, which has great potential applications in chemosensing and bioimaging.

## 1. Introduction

As sustainable materials obtained from renewable sources, cellulose nanocrystals (CNCs) have drawn greater attention during the past few decades because of their uniform nanorod-like shape, excellent mechanical properties, high specific surface areas, large aspect ratios, sustainability, biocompatibility, biodegradability, and low cost as a well-defined nanomaterial [1,2,3,4]. There are numerous active hydroxyl groups on CNC surfaces, which indicates that CNCs can be functionalized through chemical modification. Through covalent modification, fluorescent dye-labeled CNCs have been reported for fluorescence sensing, bioimaging, and drug delivery [5]. Nielsen et al. reported dual fluorescent labeling of CNCs that had pH-sensitive properties and could be used as sensing nanomaterials [6]. Huang et al. reported a mild approach for producing pH-sensitive fluorescent CNCs by labeling dye-containing groups with L-leucine amino acids [7]. Dong et al. reported fluorescein isothiocyanate (FITC)-labeled CNCs for bioimaging [8], and the in vitro study showed that folic acid (FA)-conjugated FITC-labeled CNCs selectively targeted folate receptor-positive cancer cells and were promising for cancer treatment [9]. Usually, fluorescently labeled CNCs are designed by using dyes, including fluorescein [6,7,8,9,10], rhodamine [6,7,8,9,10], quinolone [11], methylcoumarin [12], pyrene [13], and Alexa Fluor [14]. Unfortunately, as far as we know, conventional organic dyes and fluorophores are quenched at a high concentration or in a solid state, which is known as the aggregation-caused quenching (ACQ) effect. As a result, the ACQ effect significantly limits the applications of conventional fluorophores [15,16]. In contrast, aggregation-induced emission (AIE) fluorophores show luminous properties opposite to the common ACQ effect [17]. An AIE fluorogen is nonemissive when molecularly dissolved, but appears as strong fluorescence in an aggregated state [18,19,20]. 

The detection of explosives has become increasingly important due to antiterrorism applications in national security, public safety, and environmental protection [21,22]. As far as we know, nitroaromatic compounds are widely used as explosives. To date, there has been great interest in detecting trace nitroexplosives. Currently, methods for nitroexplosive detection include chemosensors [23,24], electrochemical sensors [25], mass spectrometry [26], and optical sensors [21]. Fluorescent-based sensors have been researched extensively due to their simplicity, rapid response, and excellent selectivity and sensitivity. Tetraphenylethylene (TPE) derivatives are a kind of AIE molecule that can be used for explosives detection [27]. As an electron donor, electron-rich TPE is promising for nitroexplosive detection due to good electron acceptor capacity, which lowers the energy of the empty π* orbitals of TPE, resulting in fluorescence quenching [28]. Therefore, the quenched fluorescence makes possible the detection of nitroexplosives. Feng et al. have reported that a TPE Schiff base macrocycle with AIE effects showed a selective and sensitive response to picric acid (PA) and 2,4-dinitro-phenol (DNP) in the presence of a number of nitroaromatic compounds [29]. Mahendran et al. have reported that donor–acceptor-conjugated TPE-2-pyrone (TPEP) with AIE effects showed a selective and sensitive response to PA along with a number of nitroaromatic compounds [30]. Tang et al. have reported AIE polymers with through-space conjugated effects that could serve as highly selective and sensitive sensors for detecting PA, as they showed a superamplification effect in aqueous media [31]. However, many nitrophenol-selective fluorescent probes are hydrophobic, which limits their use in aqueous systems, or are environmentally unfriendly due to being derived from petrochemicals. In this work, AIE-active CNCs (coded as TPE-CNCs) were synthesized by attaching TPE onto CNCs. The structure and fluorescent properties of TPE-CNCs were studied. Furthermore, selective detection of nitrophenolic explosives in an aqueous system were also tested. The results indicated that TPE-CNCs are excellent sensors of nitrophenolic explosives in aqueous solutions, which has promising applications for bioimaging and chemosensing.

## 2. Materials and Methods

### 2.1. Materials

Cellulose (cotton linter pulp) was purchased from Hubei Chemical Fiber Group Co., Ltd. (Xiangyang, China). Bromotriphenylethylene, 4-carboxyphenylboronic acid, trinitrotoluene (TNT)-methanol (1 mg/mL), and 2,4-dinitrotoluene (DNT) were purchased from the Aladdin Industrial Co. (City Industry, Ontario, CA, USA). PA was supplied by Xilong Chemical Co., Ltd. (Guangzhou, China). Epichlorohydrin, nitrobenzene (NB), NP, DNP, *p*-nitrotoluene (NT), and other reagents of analytical grade were purchased from Sinopharm Chemical Reagent Co., Ltd. (Shanghai, China) and were used without further purification. 

### 2.2. Preparation of CNC-NH_2_

Following Reference [9], CNCs were obtained via acid hydrolysis of cotton linter pulp. Cellulose (10 g) was added into sulfuric acid (150 mL, 64 wt %) under vigorous stirring at 60 °C. The reaction was quenched with 300 mL of deionized water after 30 min. Then, the suspension was subsequently centrifuged for 10 min at 8000 rpm, followed by washing with water until a neutral pH. Then, the suspension was dialyzed for 5 days, replacing deionized water at preset intervals. Finally, the remaining aggregates in the suspension were removed by filtration. The obtained CNC suspension was concentrated to ~2 wt % by rotary evaporator and was sonicated at room temperature for 30 min. The final CNC aqueous suspension was preserved at 4 °C within a refrigerator.

NaOH aq (50% w/v, 40 mL) was added slowly to 100 g of CNC suspension (1 wt %) under stirring and ice bath cooling. Under vigorous stirring, an excess of epichlorohydrin (0.8 g) was added, and then the suspension was stirred at 60 °C for 3 h. Then the mixture was dialyzed against deionized water until the pH was around 12. In order to introduce primary amine groups, epoxy rings were opened with ammonium hydroxide. The pH value was adjusted to 12 with NaOH aq, and then an excess of ammonium hydroxide (6 mL) was added. The reaction mixture was stirred at 60 °C for 2 h. The suspension was then dialyzed against deionized water until the pH value was constant, and the CNC-NH_2_ aqueous suspension was concentrated to 1 wt %.

### 2.3. Preparation of TPE-Labeled CNCs

**Figure 1:**
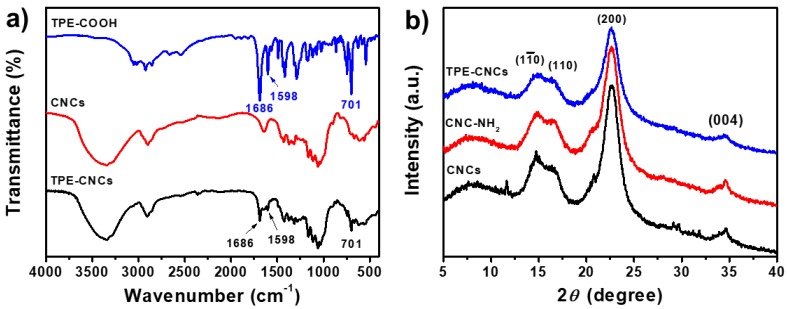
(**a**) FT-IR spectra of CNCs, TPE-COOH, and TPE-CNCs; (**b**) XRD patterns of CNCs, CNC-NH_2_, and TPE-CNCs.

TPE-COOH was synthesized according to Li et al. [32], and the analytical data were the following: ^1^H NMR (deuterium dimethyl sulfoxide (DMSO-*d*_6_), 500 MHz): δ (tetramethylsilane (TMS), ppm) 7.72–7.68 (d, 2 H), 7.20–7.12 (m, 11 H), and 7.04–6.98 (m, 6 H) (Figure S1); ^13^C NMR (DMSO-*d*_6_, 100 MHz): δ (TMS, ppm) 166.98, 147.79, 142.69, 142.57, 141.95, 141.75, 139.67, 130.71, 130.54, 130.31, 128.76, 128.58, 127.88, 127.77, 126.69, and 126.79 (Figure S1); FT-IR (KBr) *v* (cm^−1^): 698, 865, 1018, 1071, 1113, 1292, 1417, 1489, 1560, 1602, and 1690 (Figure 1a); and electrospray ionization mass spectrometry (ESI-MS): *m*/*z*: 376.2 calcd for C_27_H_20_O_2_ 376.15 [M]^+^ (Figure S2). 

Freeze-dried CNC-NH_2_ (1 g) was dispersed into DMSO (80 mL) by stirring and mild sonication. 1-(3-Dimethylaminopropyl)-3-ethylcarbodiimide hydrochloride (EDC, 0.5 g), 4-dimethylamino- pyridine (DMAP, 0.2 g), and TPE-COOH (0.5 g) were gradually added and then stirring for 1 day at room temperature. At the end of the reaction, the suspension was dialyzed against deionized water for 5 days until the dialysis water showed no fluorescence emission. The resultant suspension was concentrated and sonicated and stored in a refrigerator at 4 °C before measurements.

### 2.4. Characterizations

NMR measurement of TPE-COOH in DMSO-*d*_6_ was carried out on a Varian INOVA-600 spectrometer in the proton noise–decoupling mode with a standard 5-mm probe at 25 °C. FT-IR spectra were measured on a Nicolet 170SX FT-IR spectrometer (Nicolet Instrument Corp., Madison, WI, USA). The spectra were collected at a resolution of 4 cm^−1^, with 32 scans over a range of 4000–400 cm^−1^. The KBr-disc method was used to prepare test specimens. X-ray diffraction (XRD) was performed on an XRD-6000 diffractometer with a Cu-Kα radiation (1.5405 Å, 40 kV, 30 mA) source (Shimadzu, Tokyo, Japan). The scan range (2*θ*) was from 5 to 40°. Origin® software was used to smooth the data and correct the baseline. The degree of crystallinity of the sample was determined according to the equation [33]

$$CrI=\frac{(I_{200}-I_{am)}}{I_{200}}\times 100,$$

where *CrI* is the crystallinity index of the sample; *I*_200_ is the maximum intensity of the (200) plane of cellulose *I*; and *I*_am_ is the reflection intensity at 2*θ* = 18°, taken as the average intensity over the range 17.9−18.1°. The crystallite size of CNC was estimated by the Scherrer equation [34],

$$D=\frac{0.89\lambda }{\beta \cos\theta },$$

where *D* is the crystallite size, *λ* is the X-ray wavelength (nm), *θ* is the diffraction angle for the (200) plane, and *β* is the angular width at half-maximum intensity.

The elemental composition of the samples was analyzed using a Vario EL cube CHONS elemental analyzer (Elementar, Germany). Atomic force microscope (AFM) images were obtained in a dynamic force mode (Cypher S) using an AC240TS tip (tip radius < 10 nm, spring constant 42 N m^−1^, resonance frequency 300 kHz). Transmission electron microscopic (TEM) images were performed on an electron microscope (HR JEM-2100, JEOL, Tokyo, Japan) with an accelerating voltage of 200 kV. TEM samples were prepared by dropping aqueous suspensions onto copper grids coated with Formvar films until the solvents were evaporated in dust-protected atmosphere. Dynamic laser light scattering (DLS) was conducted on an ALV/DLS/SLS-5000E light scattering goniometer (ALV/CGS-8F, ALV GmbH, Hessen, Germany) at 25 °C to obtain the hydrodynamic radius (*R_h_*) of the samples in water. The aqueous suspensions were filtered into the light-scattering bottles through a 0.22 filter (NYL, Whatman, UK) before measurement, and the scattering angle (θ) was 90°. The ζ-potential of the suspensions was measured on a Nano-ZS (ZEN 3600) instrument (Malvern Instruments Ltd, Worcestershire, UK) at 25 °C.

### 2.5. Fluorescence Determination of TPE-CNCs

Fluorescence experiments were performed by using a spectrofluorophotometer (RF-5300pc, Shimadzu, Japan) with an excitation wavelength of 330 nm. An important parameter of fluorescent materials, fluorescence quantum yield (QY) was used to quantitatively evaluate the fluorescent intensity. In this work, the QY of TPE-CNCs in aqueous suspension was determined by a well-established reference method (quinine sulfate in 0.05 M H_2_SO_4_; literature QY: 54%) [35],

*Φ*_X_ = *Φ*_ST_(*Grad*_X_/*Grad*_ST_) (*η*^2^_X_/*η*^2^_ST_),

where *Φ* is the QY; subscripts “ST” and “X” denote standards with known QYs and TPE-CNCs, respectively; *Grad* is the gradient from the plot of the integrated fluorescence intensity versus absorbance; and *η* is the refractive index of the solvent (*η*_water_ = 1.33). In order to minimize the reabsorption effect, the absorption value at the excitation wavelength should not exceed 0.05. The QY of TPE-CNCs powders was measured on an absolute photoluminescence quantum yield measurement system (Nanolog® FluoroLog-3-2-iHR320, Horiba Jobin Yvon Ltd., Kyoto, Japan) with an F-3018 integral sphere.

Luminescence lifetimes of TPE-CNCs in aqueous suspensions and in a solid state were measured using an Edinburgh FLSP-920 photocounting system (Edinburgh Instruments Ltd., LivingstonUK) with a hydrogen-filled lamp as the excitation source.

### 2.6. Detection of Explosives

TPE-CNCs aqueous suspension (4.0 mL, 0.05 wt %) was placed in a quartz cuvette. The fluorescence quenching of the TPE-CNCs suspension was observed upon successive additions of aliquots of analyte solution. After adding the analyte, the fluorescence spectra were measured immediately with 5-nm slit widths and a 330-nm excitation wavelength. The explosive sensing performance was investigated by recording the fluorescence spectra of TPE-CNCs aqueous suspensions before and after the addition of explosives with concentrations ranging from 0.05 to 200 μM. Selectivity experiments were carried out using aqueous solutions of NB, NP, NT, DNP, DNT, and TNT-methanol (1 mg/mL). The final concentration of all interfering analytes was 100 μM.

## 3. Results and Discussion

### 3.1. Structure and Morphology of TPE-CNCs

**Scheme 1:**
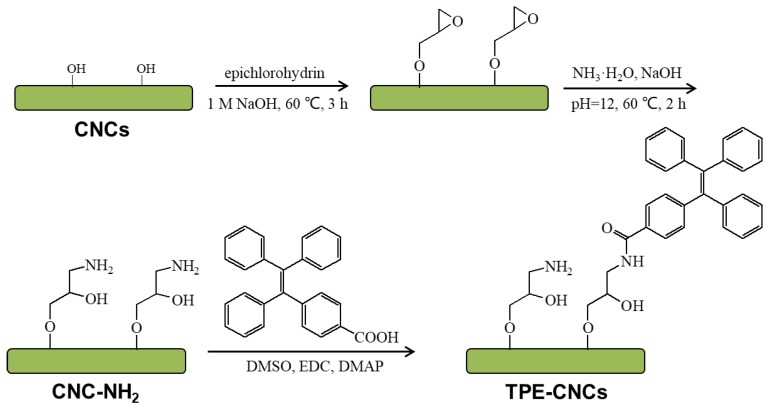
Fluorescent labeling of cellulose nanocrystals (CNCs) with tetraphenylethylene (TPE)-COOH.

Since the AIE behavior of TPE was first reported [17], various polymers composed of TPE have been synthesized [36,37,38]. As illustrated in Scheme 1, the synthesis of TPE-CNCs was performed through a substituted reaction between CNC-NH_2_ and TPE-COOH. First, CNCs were obtained by acid hydrolysis of cotton linter. Second, epoxy group-combined CNCs opened the epoxy ring to introduce amino groups for derivatization reactions [9]. Finally, TPE-CNCs were synthesized with CNC-NH_2_ and TPE-COOH. The elemental analysis results of CNCs, CNC-NH_2_, and TPE-CNCs are listed in Table S1. According to the carbon content of CNC-NH_2_ and TPE-CNCs, the TPE content on TPE-CNCs could be calculated with the following equation:

(1 × 41.79% + 27C × *m*)/[1 + (376- H_2_O) × *m*)] = 42.25%,

where *m* is the molar number of TPE-COOH reacted with 1 g of CNC-NH_2_, and 376 is the molar mass of TPE-COOH. Therefore, the TPE content was determined to be 0.027 mmol/g of cellulose (4.3 TPE moieties per 1000 anhydroglucose units). The degree of labeling calculated in this work was close to that reported by Roman and Dong (FITC content of 0.03 mmol/g of cellulose) [8].

FT-IR spectra of TPE-COOH, CNCs, and TPE-CNCs are shown in Figure 1a. For CNCs, the peaks at 1030 (C-O), 1388 (CH_2_), 2900 (C-H), and 3350 cm^−1^ (O-H) were associated with native cellulose [39]. Compared to the spectrum of CNCs, a new absorption peak at 1686 cm^−1^ appeared (corresponding to the stretching vibrations of carbonyl groups (C=O)) and was clearly detected in the IR spectrum of TPE-CNCs. Moreover, characteristic peaks at 1598 and 701 cm^−1^ (assigned to phenyl group vibrations) were observed, which demonstrated the successful introduction of TPE groups into CNCs. XRD patterns of CNCs, CNC-NH_2_, and TPE-CNCs are shown in Figure 1b. Three kinds of nanocrystals displayed characteristic diffraction peaks at 2*θ* = 14.8°, 16.5°, 22.6°, and 34.6°, which corresponded to the (1$\bar{1}$0), (110), (200), and (004) planes of cellulose I [1,4], respectively. The crystallinity index of CNCs, CNC-NH_2_, and TPE-CNCs were determined to be 87%, 82%, and 77%, respectively. These were slightly decreased due to the introduction of TPE. The crystallite sizes of CNCs, CNC-NH_2_, and TPE-CNCs were calculated to be 3.96, 3.96, and 4.13 nm, respectively. It was indicated that the chemical modification did not significantly affect their crystallite dimensions.

**Figure 2:**
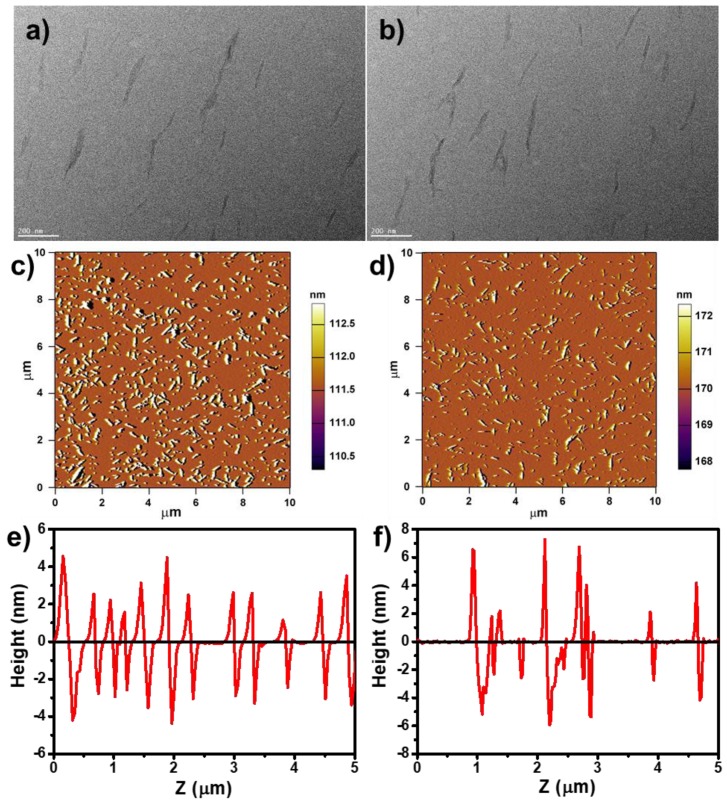
TEM images of (**a**) CNCs and (**b**) TPE-CNCs; atomic force microscope (AFM) images and height profiles of (**c**,**e**) CNCs and (**d**,**f**) TPE-CNCs.

Figure 2a,b shows TEM images of CNCs and TPE-CNCs. Both CNCs and TPE-CNCs had rod-like shapes with 20–30 nm diameters and 200–400 nm lengths, respectively. Introducing fluorophores did not destroy the basic properties and the rod-like morphology of CNCs. AFM images and height profiles of CNCs and TPE-CNCs are displayed in Figure 2c–f, and the related length and width distribution histograms are shown in Figure S3. The mean values of the length (*L*) and width (*W*) for CNCs were evaluated to be 253 ± 71 nm and 30 ± 7 nm, respectively, and the average aspect ratio (*L*/*W*) was nearly 8.4. For the TPE-CNCs, the mean values of *L* and *W* were determined to be 273 ± 92 nm and 35 ± 10 nm, respectively, and the *L*/*W* was about 7.8. The size of the TPE-CNCs was slightly larger than the CNCs due to the introduction of TPE groups. The hydrodynamic radius (*R_h_*) distributions of CNCs and TPE-CNCs in aqueous suspensions are shown in Figure S4. The *R_h_* values of the CNCs and TPE-CNCs determined by DLS were 57.9 and 86.8 nm, respectively. The ζ-potential value of CNCs in an aqueous suspension was −36.1 mV, which increased to −30.8 mV after amination. After labeling TPE, the ζ-potential value of TPE-CNCs further increased to −27.3 mV. TPE-CNCs were well dispersed in distilled water, suggesting that the modification of CNCs by TPE-COOH did not significantly affect its redispersibility in water. However, slightly increased aggregations of TPE-CNCs probably occurred in aqueous suspensions due to the change in surface charge with the introduction of TPE.

### 3.2. Fluorescence Properties of TPE-CNCs

**Figure 3:**
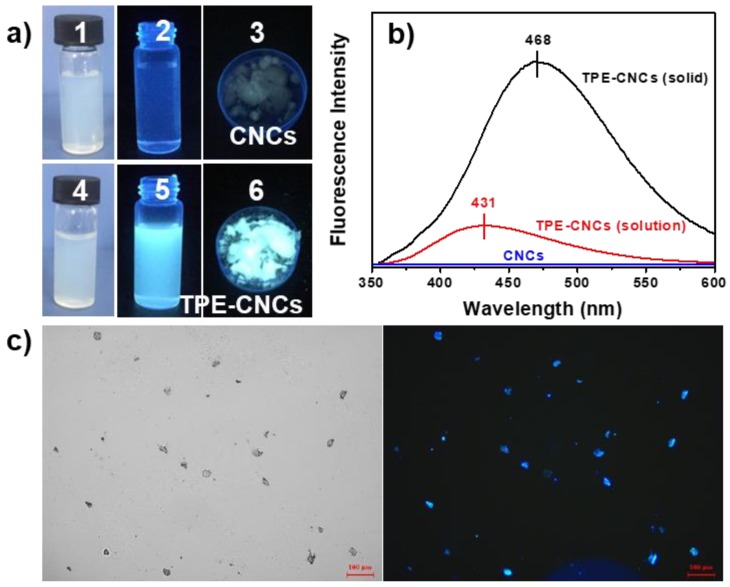
(**a**) Photographs of the aqueous suspensions of CNCs (1, 2) and TPE-CNCs (4, 5) taken under room lighting and UV illumination, and solid powders of CNCs (3) and TPE-CNCs (6) taken under UV illumination; (**b**) fluorescence emission spectroscopy of CNCs before and after modification (λ_ex_ = 330 nm and ex/em = 5/5); (**c**) fluorescent-inverted microscopic image of TPE-CNCs and a corresponding bright-field image.

Like many other TPE-modified polymers in the previous work, the TPE-CNCs were AIE-active polymers [38]. As shown in Figure 3a, TPE-CNCs aqueous suspensions were of a white colloidal solution, were slightly opaque in appearance under visible light, and emitted a strong white-blue light under UV light (365 nm). Solid powders of TPE-CNCs were gray in appearance under visible light and also emitted a strong white-blue light under UV light (365 nm). This was quite different from the fluorescence properties of the conventional ACQ fluorophores, which are usually nonfluorescent in a solid state under UV excitation due to severe self-quenching effects in the aggregated state. For example, FITC, a fluorescein dye that is reactive toward an amino group in a biopolymer, was nonfluorescent under UV illumination in the solid state because of severe self-quenching effects [40]. CNC suspensions did not show any emission peaks over the range of 350–600 nm in the fluorescent spectrum. TPE-CNCs aqueous suspensions exhibited a strong blue photoluminescence peak at 431 nm, whereas the solid powder radiated in a red-shifted wavelength at 468 nm (Figure 3b). Red shifts have also been observed in other AIE-active TPE derivatives because of the aggregate effect [40,41]. Fluorescent-inverted microscopic images also suggested the strong fluorescence emission of TPE-CNCs particles (Figure 3c).

**Figure 4:**
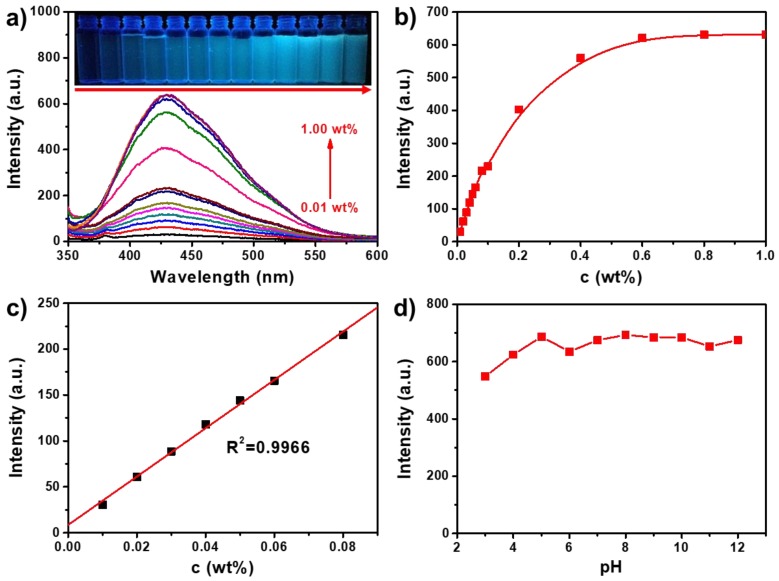
(**a**) Fluorescence emission spectra of TPE-CNCs aqueous suspensions with various contents (0.01–1.00 wt %, λex = 330 nm, and ex/em = 3/3). Inset are the photos of TPE-CNCs suspensions with various contents (0.01–1 wt %) under irradiation at 365 nm. (**b**) Dependence of the fluorescent intensity at 431 nm on the TPE-CNCs content of the aqueous suspensions. (**c**) The linearity of the fluorescent intensity at 431 nm of the suspensions with TPE-CNCs contents from 0 to 0.08%. (**d**) Dependence of the fluorescence intensity at 431 nm on the pH of the TPE-CNCs aqueous suspensions (0.05 wt %, λ_ex_ = 330 nm, and ex/em = 5/5).

The fluorescence emission spectra of TPE-CNCs aqueous suspensions with various contents and the dependence of fluorescence intensity at 431 nm on the TPE-CNCs content are shown in Figure 4a,b. A good linear relationship was observed between the fluorescence intensity of a TPE-CNCs suspension and its content over the range of 0–0.08 wt % (*R*^2^ = 0.9966; Figure 4c). When the concentration of a TPE-CNCs aqueous suspension increased from 0 to 0.6 wt %, the fluorescence intensity leveled off and hardly changed over that. The unique structure of CNCs provided a larger specific surface area and more surface hydroxyl groups. CNCs have a tendency to aggregate because of the strong interactions between their surface hydroxyl groups [2,4]. The higher the CNCs content in a suspension is, the higher the extent of the aggregation. The dependence of the fluorescence intensity on the pH value of TPE-CNCs aqueous suspensions (0.05 wt %) showed that the fluorescence intensity of TPE-CNCs aqueous suspensions hardly changed over a pH range of 4 to 12 (Figure 4d). Accordingly, TPE-CNCs suspensions could be used for chemosensing in the detection of specific substances because of their stable fluorescence properties.

The AIE characteristics of TPE-CNCs were further verified by examining their fluorescence QY values in solution and solid states. Quinine sulfate (0.1 M H_2_SO_4_, QY = 54%) was chosen as a standard sample to calculate the QY of TPE-CNCs aqueous suspensions. TPE-CNCs aqueous suspensions showed negligibly small QY values of 2.1% (Figure S5). However, solid-state TPE-CNCs emitted bright white-blue light (QY = 17.3%) measured from their solid powder by an integrating sphere. The luminescence lifetime of TPE-CNCs was measured, and it was found that the lifetimes in an aqueous suspension and in a solid state were 13.32 and 4.39 ns, respectively (Figure S6). These results indicated that TPE-CNCs exhibited a profoundly enhanced emission in a solid state compared to in a suspension (QY_solid_/QY_suspension_ > 8). In a study of benzene-cored luminophores by Tang’s group [42], the AIE features of TPE-CNCs were due to the restricted intramolecular rotation (RIR) process in an aggregated state, which blocked the nonradiative relaxation channel and populated radiative decay. The AIE features of TPE-CNCs were attributed to their morphology and structure. According to the RIR mechanism, the phenyl rings of TPE were still freely rotated in solution even after TPE units were incorporated into CNCs, making TPE-CNCs weakly emissive. However, when TPE-CNCs were in an aggregated state, the rotation of the phenyl rings of TPE units was restricted, which effectively blocked nonradiative energy dissipation channels of TPE-CNCs and led to highly fluorescent emission.

### 3.3. Detection of Nitrophenolic Explosives

**Figure 5:**
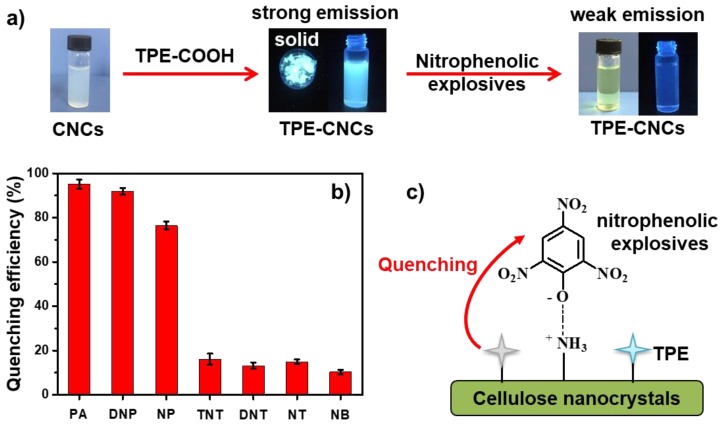
(**a**) Schematic representation and photographs of the fluorescence-labeling CNCs with TPE-COOH, and with the addition of nitrophenolic explosives; (**b**) fluorescence quenching efficiency of different nitroaromatic compounds; (**c**) schematic representation of the selective sensing and simulation results of the electrostatic interaction between nitrophenolic compounds and TPE-CNCs.

TPE derivatives present good electron donor ability, and the electron transition might occur from an electron-rich delocalization structure to electron-deficient nitroaromatics. Therefore, they have been shown to be promising sensor materials for detecting nitroaromatic explosives [43,44]. Thanks to the good dispersion of TPE-CNCs in water, they can be used to detect explosives in aqueous systems. Herein, the selectivity of this fluorescent TPE-CNCs detection system was investigated for nitroaromatic compounds, including PA, DNP, NP, TNT, DNT, NT, and NB. A TPE-CNCs aqueous suspension (0.05 wt %) was used as a probe, and an increased amount of nitroaromatic compounds were gradually added (Figure 5a). The relative fluorescence quenching efficiencies of TPE-CNCs suspensions in the presence of 100 μm of nitroaromatic compounds showed that fluorescence was much more easily quenched by nitrophenolic compounds (PA, DNP, and NP) than by other nitroaromatic compounds (Figure 5b). The fluorescence quenching efficiencies ((1 − *I*/*I*_0_) × 100%) caused by PA, DNP, NP, TNT, DNT, NT, and NB were about 95.1, 91.9, 76.4, 16.1, 13.2, 15.0, and 10.3, respectively. These results showed that TPE-CNCs had excellent selectivity toward the detection of nitrophenolic explosives over the other interfering nitroaromatic compounds. 

**Figure 6:**
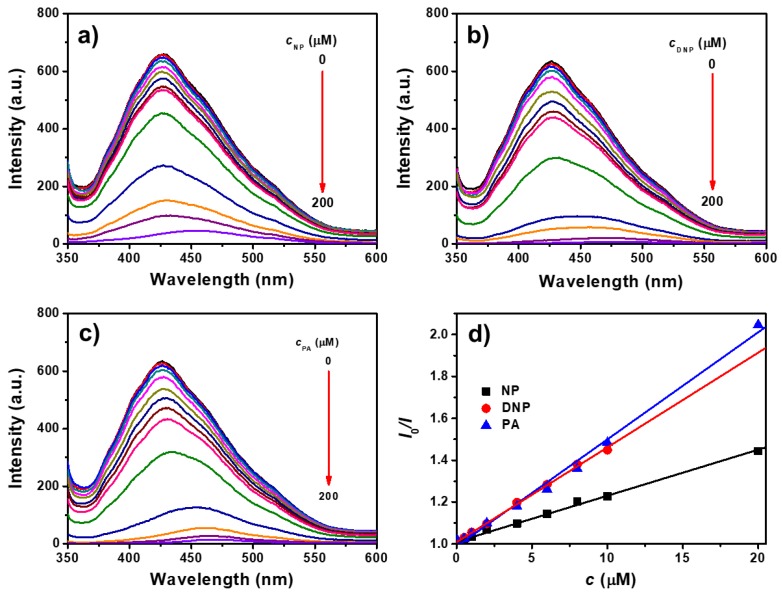
Fluorescence emission spectra of TPE-CNCs aqueous suspensions (*c*_TPE-CNCs_ = 0.05 wt %) with the addition of (**a**) 4-nitrophenol (NP), (**b**) 2,4-dinitro-phenol (DNP), and (**c**) picric acid (PA) (λ_ex_ = 330 nm, ex/em = 5/5). (**d**) Stern–Volmer plots of TPE-CNCs suspensions for NP, DNP, and PA.

The fluorescence emission spectra of TPE-CNCs aqueous suspensions with the addition of NP, DNP, and PA are shown in Figure 6a–c, respectively. The fluorescence intensities of TPE-CNCs suspensions were gradually quenched with increasing nitrophenolic explosive concentrations. The fluorescence emission was completely quenched after the addition of 200 μM of NP, DNP, or PA. In addition, fluorescence quenching was detected in short response times, and the quenching equilibrium was instantaneously established within several seconds. The Stern–Volmer equation was used to evaluate the quenching efficiency as follows [45]:

*I*_0_/*I* = 1 + *K*_SV_ × *c*,

where *I*_0_ is the initial fluorescence intensity in the absence of analyte, *I* is the fluorescence intensity in the presence of analyte concentration (*c*), and *K*_SV_ is the quenching constant. Stern–Volmer plots of the TPE-CNCs suspensions for PA, DNP, and NP showed good linear relationships in all cases for fluorescence quenching, and the *K*_SV_ was 5.1 × 10^4^, 4.5 × 10^4^, and 2.2 × 10^4^ M, respectively (Figure 6d). The *K*_SV_ calculated from the Stern–Volmer plots could also be used to indicate quenching efficiency, such that for higher values of *K*_SV_, a higher quenching efficiency was obtained. Therefore, explosives were quantitatively detected by fluorescence quenching, and the limit of detection (LOD = 3σ/*K*) was determined to be 220, 250, and 520 nM for PA, DNP, and NP, respectively. Herein, σ is the standard deviation of the blank measurements (*n* = 11), and *K* is the slope of the curve (*K*_SV_). With its high stability and sensitivity to PA, DNP, and NP, the fluorescent TEP-CNCs were observed to be good chemosensors in aqueous systems.

**Figure 7:**
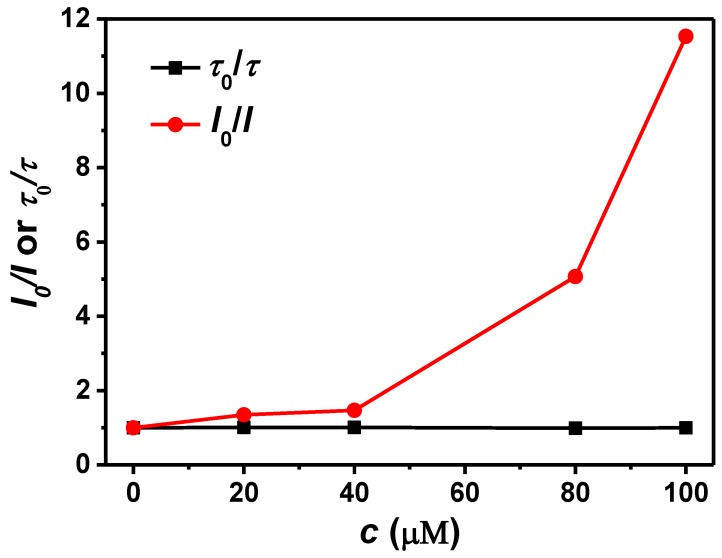
Plots of *I*_0_/*I* and *τ*_0_/*τ* of TPE-CNCs aqueous suspensions against the concentration of PA.

Like other electron-deficient nitroaromatics, PA, DNP, and NP could quench TPE-CNCs fluorescence emission via a donor–acceptor interaction mechanism because the sensing fluorophore introduced into the CNCs was an electron-rich group. However, in this study, the situation was much more complicated, because the substrate CNCs were not inert due to the presence of amino groups, and they had some interactions with the hydroxyl groups of nitrophenol. The special selectivity of TPE-CNCs for PA, DNP, and NP might be understood by considering such interactions. The possible quenching mechanism is shown in Figure 5c. It is well known that PA behaves as a strong acid due to the presence of three nitro groups on the benzene ring. In contrast, there are a large number of hydroxyl and amino groups on TPE-CNCs, which have a strong tendency to attract protons. Therefore, proton transfer occurs between PA and TPE-CNCs. This electrostatic association gives PA a special affinity for TPE-CNCs. This might be the reason why PA, DNP, and NP are so efficient at quenching fluorescence emission in TPE-CNCs suspensions. Other electron-deficient nitroaromatics, such as NB, NT, DNT, and TNT, have no hydroxyl groups, as in nitrophenol, and therefore they have no tendency to be enriched on TPE-CNCs surfaces. Furthermore, CNCs are hydrophilic nanoparticles. However, electron-deficient nitroaromatics are essentially hydrophobic. Therefore, the compatibility of substrate CNCs with these electron-deficient nitroaromatics is poor, and the substrate screens the approach of quencher molecules toward fluorescence-active TPE-CNCs, leading to lower quenching efficiencies. Fluorescence lifetimes of TPE-CNCs in aqueous suspensions with various PA concentrations were measured to further study the quenching mechanism (Figure S7). In the case of dynamic quenching, fluorescence lifetimes varied proportionally to quencher concentration. However, lifetimes were invariant under quencher concentrations with static quenching. The fluorescence decay curves were hardly changed with the addition of PA (Figure S7). Upon examination of the plots shown in Figure 7, it is clear that the lifetime of TPE-CNCs was almost independent from the concentration of the quencher, indicating that fluorescence quenching occurred through a static mechanism via the formation of a nonfluorescent complex.

## 4. Conclusions

In summary, AIE-active CNCs were synthesized, and the fluorescence properties were studied. TPE-CNCs displayed good dispersity in aqueous systems and exhibited excellent AIE optical properties due to the introduction of TPE groups. TPE-CNCs aqueous suspensions showed excellent luminescent properties and a high fluorescence quenching sensitivity to nitrophenolic compounds (NP, DNP, and PA) among a number of nitroaromatic compounds. The detection limits were determined to be 220, 250, and 520 nm for PA, DNP, and NP, respectively. The fluorescence quenching occurred through a static mechanism via the formation of a nonfluorescent complex between TPE-CNCs and nitrophenolic analytes through strong electrostatic interactions. Therefore, TPE-CNCs exhibited great potential for the development of feasible, stable, and sensitive chemosensors for detecting nitrophenolic explosives in aqueous solutions. Moreover, TPE-CNCs, as renewable, abundant, and biocompatible media, might also be expected to be useful in applications in biological sensing and imaging.

## Supplementary Materials

The following are available online at https://www.mdpi.com/2079-4991/9/5/707/s1, Figure S1: 1H and 13C-NMR spectra of TPE-COOH in DMSO-*d*_6_; Figure S2: ESI-MS spectrum of TPE-COOH; Figure S3: Size distribution histograms of CNCs and TPE-CNCs; Figure S4: Hydrodynamic radius distributions of CNCs and TPE-CNCs in aqueous suspensions; Figure S5: Integrated fluorescence intensity versus absorbance plots of TPE-CNCs and quinine sulfate; Figure S6: Fluorescence decay profile of TPE-CNCs in aqueous suspension and solid powder; Figure S7: Fluorescence decay profiles of TPE-CNCs suspensions in the presence of various PA concentrations; Table S1: Elemental analysis results of CNCs, CNC-NH_2_, and TPE-CNCs.
