# Supplementary material for: Aggregation-Induced Emission (AIE)-Labeled Cellulose Nanocrystals for the Detection of Nitrophenolic Explosives in Aqueous Solutions
Source: Nanomaterials (Basel). 2019 May 7;9(5):707. doi: 10.3390/nano9050707 (PMC6567080; doi:10.3390/nano9050707)
Supplement: Supplementary file 1 [file nanomaterials-09-00707-s001.pdf]

Supplementary Materials

Aggregation-Induced Emission (AIE)-Labeled Cellulose Nanocrystals for the Detection of Nitrophenolic Explosives in Aqueous Solutions

Xiu Ye, Haoying Wang, Lisha Yu and Jinping Zhou *

Department of Chemistry, Engineering Research Center of Natural Polymer-based Medical Materials in Hubei Province, and Key Laboratory of Biomedical Polymers of Ministry of Education, Wuhan University, Wuhan 430072, China; yx444131997@gmail.com (X.Y.); wang159hy@gmail.com (H.W.); LisaYu12@whu.edu.cn (L.Y.)

*Correspondence: zhoujp325@whu.edu.cn; Tel.: +86-27-68752977 (J.Z.)

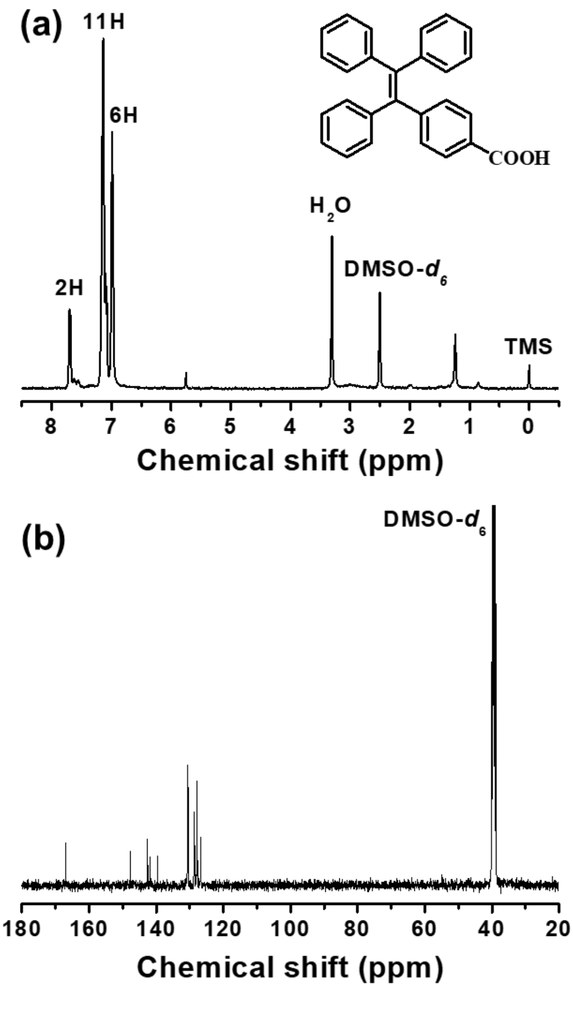

Figure S1. (**a**) ^{1}H and (**b**) ^{13}C NMR spectra of TPE-COOH in DMSO-*d6* at 25 °C.

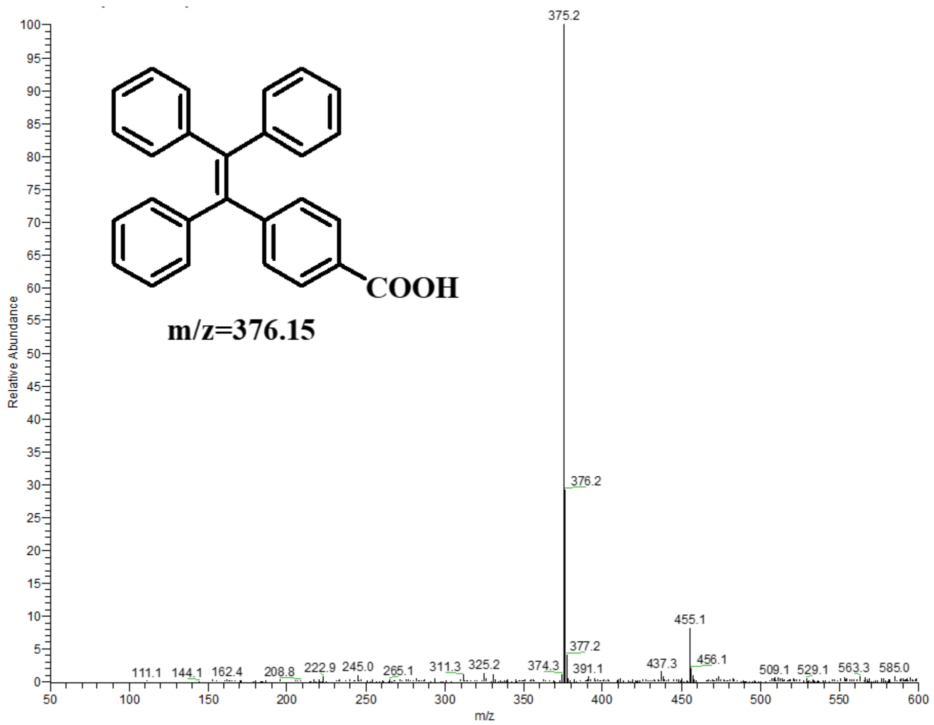

Figure S2. ESI-MS spectrum of TPE-COOH.

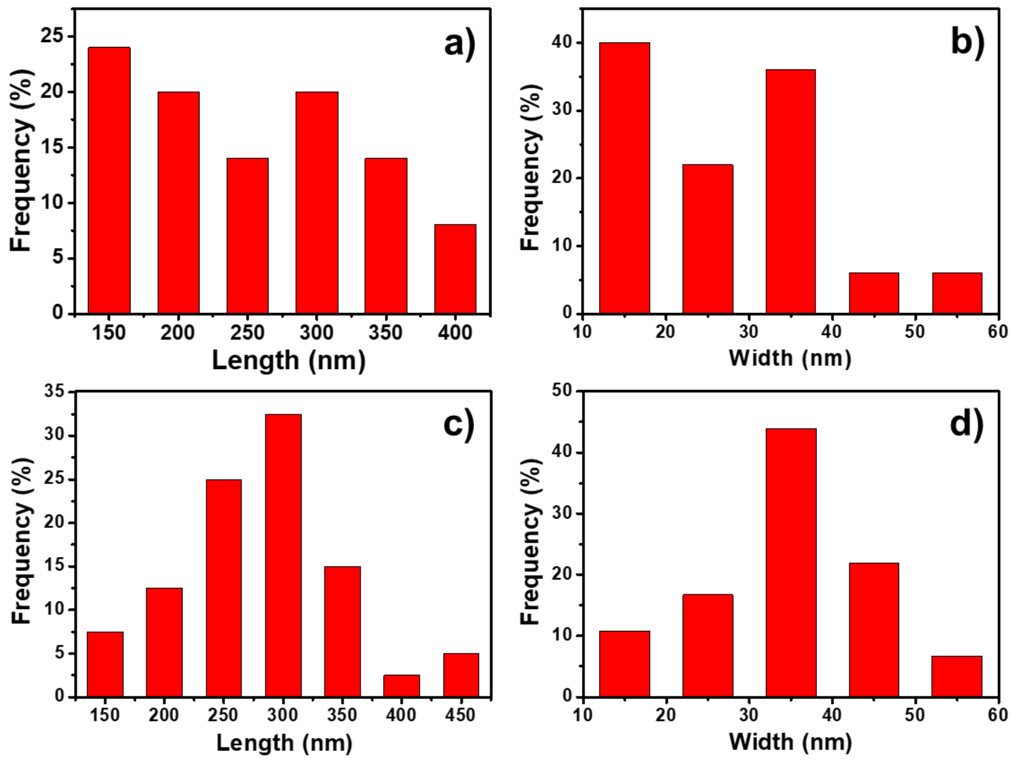

Figure S3. Size distribution histograms of (**a**,**b**) CNCs and (**c**,**d**) TPE-CNCs calculated from AFM images.

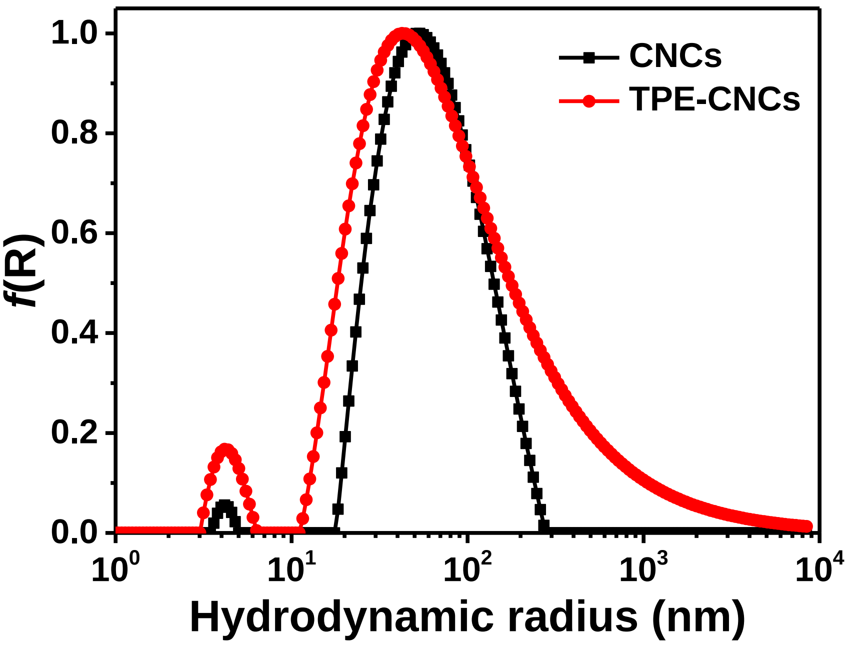

Figure S4. Hydrodynamic radius (R_h) distributions of CNCs and TPE-CNCs in aqueous suspensions.

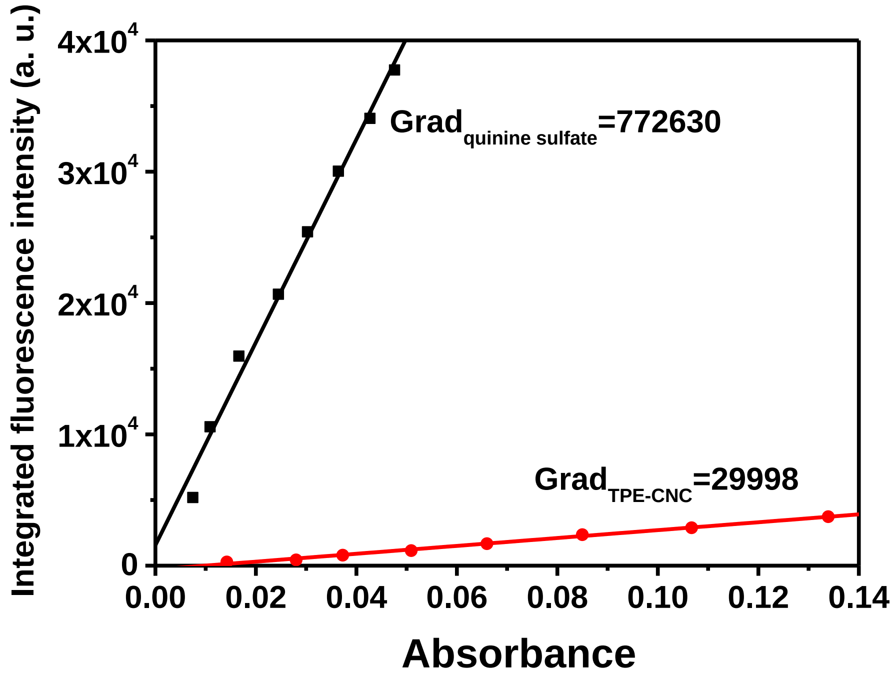

Figure S5. Integrated fluorescence intensity versus absorbance plots of TPE-CNCs and quinine sulfate.

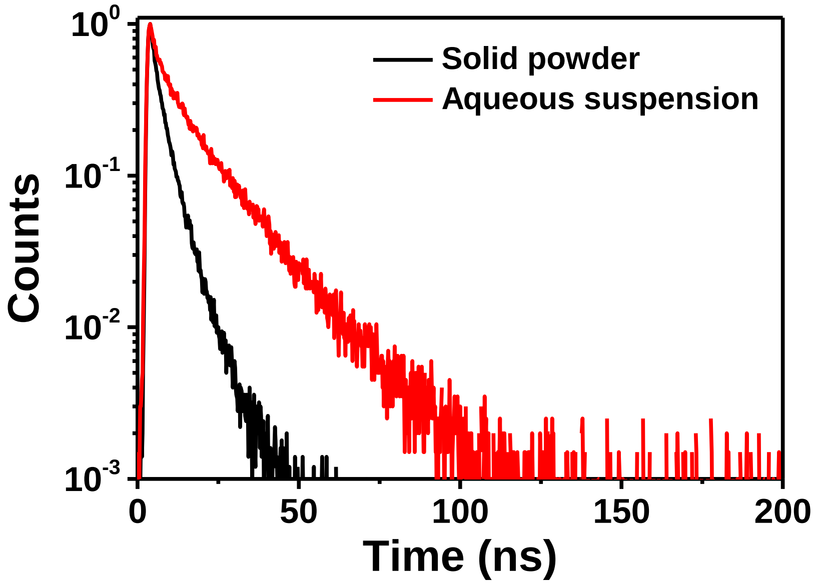

Figure S6. Fluorescence decay profiles (λ_{em} = 431 and 468 nm) of TPE-CNCs in aqueous suspension and solid powder.

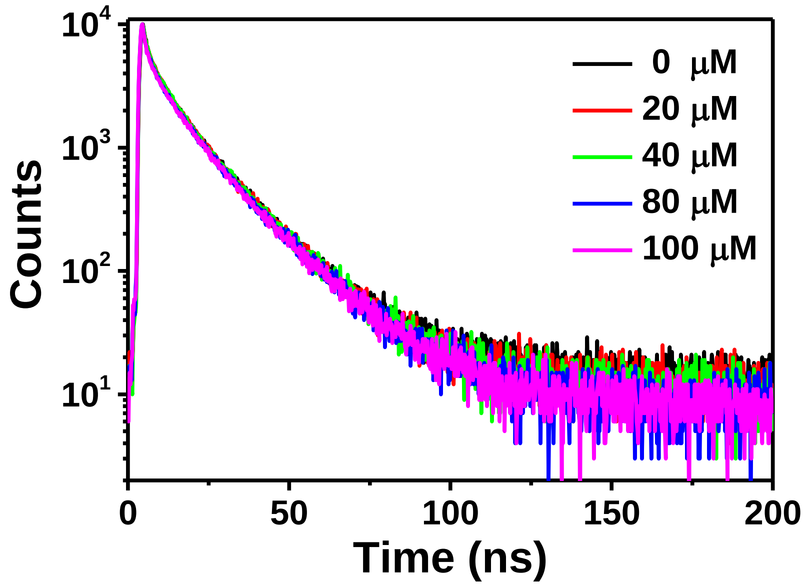

Figure S7. Fluorescence decay profiles (λ_{em}=431 nm) of TPE-CNCs aqueous suspensions in the presence of various PA concentrations.

Table S1. Elemental analysis results of CNCs, $CNC-NH_2$ and TPE-CNCs.

Sample	C (wt %)	O (wt %)	H (wt %)	N (wt %)
CNCs	41.75	48.66	6.32	0
$CNC-NH_2$	41.79	49.00	6.22	0.18
TPE-CNCs	42.25	48.73	6.65	0.37
